# Robot as Legal Person: Electronic Personhood in Robotics and Artificial Intelligence

**DOI:** 10.3389/frobt.2021.789327

**Published:** 2021-12-23

**Authors:** Sergio M. C. Avila Negri

**Affiliations:** Department of Private Law, Federal University of Juiz de Fora, Juiz de Fora, Brazil

**Keywords:** artificial intelligence, legal personhood, robotics, electronic personhood, legal person

## Abstract

This paper seeks to investigate the proposal to create a legal (electronic) personhood for robots with artificial intelligence based on the European Parliament resolution with recommendations on Civil Law and Robotics. To this end, we highlight the various risks and problems present in this type of initiative, especially in view of the current trend of expanding legal subjectivity in various jurisdictions. In addition to an anthropomorphic rhetoric, we can observe the prevalence of a pragmatic line that seeks to be guided, mainly, by the model of corporations, without taking into account, however, problems present in the process of embodiment of companies and the particular function of the term legal person in the grammar of Law.

## Introduction

In his essay *The Sphere of Pascal*, the writer Jorge Luis Borges reports that the Greek philosopher Xenophanes, master of Parmenides, was tired of the Homeric verses that dressed the Gods as human beings. In opposition to anthropomorphic traits, he proposed to the Greeks one God, who was in fact an eternal sphere. History followed its course and the exaggeratedly human gods were relegated to poetic fictions.

The anthropomorphic metaphor is not restricted to mythical or religious imagery. Sophia, a humanoid robot with Artificial Intelligence (AI), developed by the Hanson Robotics company, received citizenship from Saudi Arabia in 2017. Although several interviewers were impressed with the sophistication of its responses, the robot follows a simple algorithm and most of its statements are credited to a previously prepared text (Parviainen and Coeckelbergh, 2020).

As in Borges’ essay, Robotics can also be thought of without any anthropomorphic resource, with other metaphors, as a sphere. Roomba is a flat, round domestic robot. Even though it does not have social skills like Sophia, the fact that this robotic vacuum cleaner moves on its own, following a simple algorithm, causes some people to give it a name, talk to it and feel bad when the appliance gets stuck under the sofa (Darling, 2016).

If, for a long time, the idea that robots and human beings should be separated was in force, an opposite trend has been accentuated, especially in the last decade: human beings can and should share the same environment as robotic artefacts. As escorts of the elderly—and even children with autism—surgical apparatus, deliverers or security guards, robots have already begun to enter people’s homes and lives.

Because of the lack of ontological and legal definition about this emerging technology, the Law is forced to resort to old figures, already-known metaphors, which help us to approach with a certain familiarity what is new and unknown. In 2017, the European Parliament put forward a resolution with guidelines on Robotics, with a proposal to create an electronic personhood for “intelligent” robotic artefacts (European Union, 2017).

In the verbalized legal world, the term “legal person” refers to an autonomous centre of legal relations. The ascription of legal personhood is based on the assumptions that all legal relations take place among natural person and artificial legal person, such as corporations. Following that, the term natural person refers to a human being. By contrast, the term “legal person” or “legal entity” will be often used in this paper when referring to the artificial legal person.

According to Gurnkel (2018a, 2018b), it is important to separate certain questions that are confused in the debates about the legal personhood of robots. First, there is a relevant difference between the two verbs that comprise the question: “can” and “should” AI be persons (Gurnkel, 2018a.) On the other hand, there is another relevant difference, between natural person and legal person. Following that, if legal personhood is already dissociated from the human substrate, there would be no way to deny that AI can be a legal person. But, just because it is possible, that does not mean it should be a good idea.

Just as it is important to separate the idea of moral personhood from the concept of legal personality, it should also be noted that the moral community is not limited to the figure of moral agents, currently reaching the figure of moral patients, who are affected by the actions of (“rational”) agents. This means that rights must not be confused with moral personhood. Likewise, courts might recognize certain legal rights without this implying the recognition of a moral personhood or a general legal personality.

In dialogue with these important elements of the debate on the legal personality of robots, I would like to highlight in this work a distinct aspect, very sensitive to the practice of Law: the legal entity presents itself as a decision structure, which allows the identification of problems and normative solutions used in previous cases. In this sense, it is important to understand the heuristic function of the term legal entity, that is, a mental shortcut that allows, with simplified information, quick judgments.

In the debate on electronic personhood, it is commonly observed that the legal person is presented as if there were no problems in the process of attributing legal personhood to corporations and companies. The analogy with the legal person requires, however, an understanding of the function of this term in legal grammar. This paper seeks to investigate the proposal to create a legal (electronic) personhood for robots based on the European Parliament resolution with recommendations on Civil Law and Robotics. To this end, we highlight the various risks and problems present in this type of initiative, especially in view of the current trend of expanding legal subjectivity in various jurisdictions. In addition to an anthropomorphic rhetoric, we can observe the prevalence of a pragmatic line that seeks to be guided, mainly, by the model of corporations, without taking into account, however, problems present in the process of embodiment of companies and the particular function of the term legal person in the grammar of Law.

## Private Law and Robotics

The architecture of digital platforms is capable, in certain cases, of influencing society more directly and efficiently than Law itself. In the growing scenario of technical regulation, it is important to note that programmers and engineers may have difficulty translating ethical and fundamental values into demands that decisively affect people’s lives. In this sense, Langdon Winner (1985), as Leenes (2011) recalls, was already working with the political dimension of artefacts and cited, for example, the absurd, structurally elitist urban constructions of Robert Moses in New York, which were designed to physically impede the passage of public transport to noble areas of the city, since it was predominantly used by the black population.

With the emergence of cyberspace, Information and Communication Technologies (ICTs) have come to be understood as instruments capable of conditioning behaviours. The relationship between Law and the normative effects of technology has been consolidated as a field of study. Lessig (2006) presents the “code”—in his words, the hardware and software that make up cyberspace—as a new form of regulation, since it defines the terms in which interactions in cyberspace take place. Thus, as the code changes, so does the character of cyberspace. Technology always incorporates certain rules, which allow a certain behaviour and inhibit another. Therefore, the rules in cyberspace are increasingly shaped by technology rather than by Law.

Robotics cannot be seen as a novelty. In industry, with emphasis on automobile manufacturing, Robotics represents a technique already incorporated into production, mainly in relation to the performance of routine tasks. As Pagallo (2018) points out, more than 50 years ago “robots have already materialised as a reprogrammable machine, operating semi or fully automatically in manufacturing operations and other industrial tasks” (Pagallo, 2018). Although Robotics should not be confused with AI, it is undeniable that today these fields are more and more closely intertwined, mainly due to the improvement of probabilistic methods, the increasing availability of huge amounts of data and the increase in computational power. One cannot forget, either, the more recent transformation of places and spaces into environments more receptive to information technology, as occurs with the imagery of intelligent cities.

The European Parliament Resolution of February 16, 2017 established that a robot shall be considered intelligent when it has the following characteristics: 1) the existence of sensors capable of allowing it to exchange data with the environment; 2) the ability to learn from experience and interact with the environment; 3) the existence of material support; 4) the ability to adapt and 5) the absence of life in the biological sense (European Union, 2017).

Among the recommendations on the constitution of a suitable registry, the formation of insurance schemes and compensation funds is the suggestion of the creation of a legal status of robots for more complex artefacts, which would then be endowed with a legal (electronic) personhood.

Electronic personhood is presented as an answer to the problems of liability in view of possible damage that could be caused by robotic artefacts. Indeed, we can discern some confusion in this form of approach: the attribution of a supposed legal personhood for robots is treated as an automatic consequence of the debate on liability. As Pagallo (2018) noted, just as we should not confuse apples with oranges, it is important to separate apples from liability and oranges from personhood. In addition to polarising the debates, it should be noted that the defence or criticism of legal personhood for robots necessarily involves an understanding of the process of conferring legal personhood on business companies and corporations. If the law does not restrict the attribution of legal personhood to human beings, how could we criticize the attribution of legal personhood to a robot ? Should we approach robotic artefacts by means of old categories, as if robots were people for the law?

## The Android Fallacy and Anthropomorphic Rhetoric

### Robots as Natural Persons

Tracing the relationship between Law and new technologies is not an easy task and, generally, this harmonisation does not occur in a simple way. This link is often made possible through the use of metaphors, which serve as an instrument for the achievement of a rhetorical effect, directly comparing different concepts. Richards and Smart (2013) explain that, when dealing with different types of robots, there are a series of competing metaphors, so choosing which ones to use generates consequences of great importance for the success or failure of an attempt to regulate Robotics.

Calo (2015) asserts that currently we are already dealing with the choice of metaphors for robots, as drones have already been equated with “aircraft”, leading to severe limitations of usage. In addition, regulatory agencies in the United States have already compared surgical robots to laparoscopic surgery, which is minimally invasive, speeding up the process of approval.

A particularly seductive metaphor, not only for the law but also for other fields of study of Robotics, is to think of robots based on anthropomorphic rhetoric, as if they were people. If the imagery about robots is marked by the presence of anthropomorphic artefacts, such as the androids of films and literature, what would be the problem for the law to resort to this subtle comparison as well? To understand the risks of this rhetoric, which projects human qualities on robots with AI, we need, first, to better understand this technology.

Faced with the challenges brought by the spread of intelligent robots, which are gradually coming onto the market and consequently are becoming more and more present in people’s lives, also impacting the sphere of Law, Calo (2015) presents three distinctive characteristics of robots: embodiment, emergence and social meaning. One of the main characteristics of a robot is to be physically incorporated into the world, which allows it to share the physical environment with human beings. As Mataric (2014) points out, corporeality also means perceiving other bodies and objects around it, because one of the first things a robot must internalize when programmed is how to avoid collisions, which is done with the help of sensors, physical devices that allow a robot to receive information about itself and the objects around it. In this sense, contrary to what it may seem, uncertainty is part of Robotics and arises from the fact that robots are physical mechanisms that operate in situations in which it will be difficult to know exactly their own state and that of their environment.

Materiality is not just a purely aesthetic issue. The way we think about robots (and their human operators) will also affect their design. In this context, Richards and Smart (2013) question what society expects of robots based on metaphors: are they virtual butlers, virtual pets, or virtual children? The answers chosen for these questions will affect the physical presentation of the robot and its configuration. According to Coeckelbergh (2009), ascribing responsibility to such agents is to experience, feel and perceive a form and performance. In this sense, one could speak of “virtual agency” and “virtual responsibility” to refer to “the responsibility that humans attribute to each other and to (some) non-humans based on how the other is experienced and appears to them” (Coeckelbergh, 2009).

Despite its anthropomorphic traits, Sophia, the humanoid robot, follows a simple program. On this point, the metaphor can be transmuted into a fallacy: human appearance can lead us to think of robots as people. Thus, since not all robots are androids, the illusion caused by anthropomorphism of form can be dangerous when we think of regulatory initiatives based on false assumptions about the capacity of robotic artefacts themselves.

The projection of human characteristics on robots does not depend on their form. Even when a robotic artefact has no anthropomorphic shape, people project onto these technologies human qualities such as consciousness and intelligence. As the autonomy of the system increases, making connections between the inputs (its commands) and the behaviour of the robot difficult, analogies with human beings are reinforced, which, in turn, can hinder any normative attempt, whether in terms of ethical debate, or in legal matters, such as the determination of who would be liable for possible damage caused by robotic artefacts.

### The Naturalisation of Autonomy and Consciousness in Robotics and in AI

In the debate on electronic personhood, it is commonly observed that already existing legal norms would be incapable of portraying and, consequently, disciplining autonomous, intelligent robots. Since it is admitted that today’s robots can perform unanticipated behaviour, we would only have to recognise their legal (electronic) personhood. This kind of reasoning has several flaws. The first is the lack of determination of the meaning of autonomy. At the same time, autonomy is confused with unpredictability of the result. Machines operated by direct human control can bring about unpredictable results. From a technological perspective, could the term “autonomy” be used in robotic applications where teleoperation, telepresence or human supervision are found at some point? Could a robot acting without constant human monitoring, but controlled at a time of need, be qualified as autonomous (Bertonili, 2013)? In this sense, the absence of specification of the term “autonomy” contributes to its own naturalization, that is, autonomy is presented as a given, as if it were a necessary consequence of the supposed intelligence of these systems.

In an attempt to dispel this imprecision, Bertolini (2013) highlights three meanings for the term autonomy when discussing robotic applications: 1) autonomy as consciousness or self-consciousness, which would lead us to the idea of free will and, consequently, to the identification of a moral agent; 2) the capacity to interact independently in the operational environment; 3) the capacity to learn.

In philosophical terms, autonomy, in a strong sense, is related to the idea that responsibility can only be attributed to a moral agent. Like subjectivity, autonomy, in that sense, is part of the philosophical discourse of modern times. Moral concepts in “modern times” have come to be shaped to recognize the subjective freedom of the individual in discerning as valid what they should do. By breaking with the paradigm of morality as obedience, Kant practically invented the concept of morality as autonomy (Schneewind, 1998). The rejection of the inequality of moral quality makes each one their own legislator, to the extent that every person would be capable of evaluating their own action, without the need for any external interference. Although the strong anthropocentric component of this idea of autonomy can be criticised, there is currently no robotic artefact that meets these described conditions, which would in principle rule out qualifying robots as autonomous agents in a strong sense. Since the law does not restrict legal personhood, as an aptitude to acquire duties and rights, to the human substrate, the ontological debate ends up losing space when confronted with more pragmatic arguments, such as the attribution of legal personhood to corporations and other business associations.

In another sense, autonomy could be understood as the ability to perform tasks without human supervision. This is autonomy in a weak sense. From the autonomous drone, to vehicles without a driver, to the robotic vacuum cleaner, one can speak, in these cases, of autonomy at various levels, even if the robotic artefact is associated with performing a certain activity due to a goal previously defined by a programmer. Although far from the idea of a strong agency concept, it is undeniable that this is an appearance of agency, which, as we have seen, has its importance. In the classic definition of Richards and Smart (2013), robotic artefacts are analysed from this sense of agency, which is not to be confused with its strong sense. In this aspect, a robot can be understood as a built system that displays, even if only apparently, a physical and mental agency, but is not alive in the biological sense, that is, it is something manufactured, that moves around the world (materiality), seems to make rational decisions about what to do (weak or apparent autonomy) and is a machine.

To avoid anthropomorphic rhetoric, Calo (2015) avoids the use of the term “autonomy” and prefers to use the term emergence. This behaviour is found in complex adaptive systems where there is a global behaviour resulting from individual interaction. Some examples can be seen in the animal world, such as the flock of birds, the school of fish and the swarm of bees, which show the creation of patterns without the existence of a central command. Emergent behaviour is a characteristic phenomenon of complex adaptive systems (Doneda et al., 2018). It is a type of global behaviour, which can result from hundreds and thousands of simple individual interactions. They create the illusion of central coordination. We speak of emergence when we observe a behaviour that is not explicitly programmed, but which results from the interaction of simple mechanisms.

The notion of emergence is associated with a holistic perspective, in which the robot’s behaviour is not confused with the simple sum of its parts, creating, in some situations, the sensation that the artefact performed an unexpected, non-programmed behaviour. It is interesting to realize that surprise can depend on the subjective expectation of the expecter. Even so, even if one adopts the perspective of the programmer, there is no way to establish beforehand all the behaviours that emerge from the interaction that occurs only in a certain time and space of the execution. As Mataric (2014) points out, the fact that we cannot predict everything in advance does not mean that we cannot predict anything, such as the risks associated with the use of artefacts, such as surgical robots, in a context of a particular use. Thus, the input received by the robot continues to be determinant for the behaviour it will produce, even if the latter is unexpected.

Autonomy can also be associated with a supposed ability to learn. Could the ability of a robot to acquire and elaborate data to perform its activities be equated to real learning? There are already robotic artefacts capable of deciding independently on the course of an action without any human intervention. Could the rules that determine the action and decisions be changed by the robotic artefact itself? What does this machine learning consist of? AI systems need the ability to acquire their own knowledge by extracting patterns from raw data. This resource is known as machine learning. The learning process, which may or may not be supervised, allows the system itself to do the same task more efficiently with each attempt, thus automatically improving its experience. Among the types of learning, the outstanding one today is deep learning, which attains great power and flexibility in the attempt to represent the outside world with an aligned hierarchy of concepts, allowing the classification of images, speech recognition and object detection, among other uses.

As Goodfellow et al. (2016) point out, the first deep learning algorithms we recognize today were thought of as computational models of biological learning, that is, models of how learning happens or can happen in the brain. Deep learning is closely associated with the architecture of artificial neural networks. Here it is noted that anthropomorphism is not a unique characteristic of Robotics. AI has also been historically conceptualised in anthropomorphic terms. As Watson (2019) points out, besides the fact that people always talk about machines that think and learn, the name itself (artificial intelligence) challenges us to repeatedly compare human ways of reasoning with algorithms. In the same way as with legal entities, it is not always clear whether this language is used in a literal or metaphorical sense.

The anthropomorphic metaphor conceals functional aspects of artificial intelligence, so that this rhetoric, which mimics human qualities and attributes, may compromise the response to the complex ethical challenges posed by emerging technologies. In fact, it is a mistake to suppose that these algorithms can be confused with human intelligence, since, although they surpass human intelligence in certain aspects, they also fall short in others (Watson, 2019). Even though one cannot criticize simple inspiration in human models for the development of artificial intelligence, it is always important to be careful when differences are erased and one begins to think of metaphors and analogies in their literal sense. Consequently, when thinking about any attempt to discipline or regulate Robotics, it is fundamental not to confuse the existence of real autonomy or agency with the sensation of autonomy or agency. Unfortunately, the confusion between the supposed agency of the artefacts and the sensation provoked by the emerging technology leads to a naturalization of the autonomy itself, as if every robot with AI necessarily was, as happens with human beings, making a decision in a specific and independent way.

### Social Robots, Vulnerability and Social Valence

It is important to separate certain issues that are confused in the debates about the legal personhood of robots. Anthropomorphism does not depend on the beliefs people may have about the ontological nature of artefacts. Even acknowledging that current questioning about the status of “intelligent” robots may impact on how people reflect and relate to these artefacts, the debates about the supposed agency of robots, or about the technical possibility of developing a complex artificial intelligence system, called strong AI, may not condition people’s willingness to continue to explain the behaviour of a robotic artefact based on the assignment of mental states. This happens on account of the particular social valence of this technology.

Moreover, social meaning (or social valence) relates to the fact that humans show greater social commitment and provide different stimuli when dealing with robots compared to other goods. This characteristic can be linked to embodiment, since the physical embodiment of the robot tends to make a person treat that moving object as if it were alive. This is even more observable when the robot has anthropomorphic characteristics, since the resemblance to the human body causes people to start projecting emotions, feelings of pleasure, pain and care, as well as desires to constitute relationships. Balkin (2015) understands that the projection of human emotions on inanimate objects is not a recent phenomenon in human history, but when applied to robots, it entails numerous consequences.

Calo (2015) lists some consequences that can be generated by social valence, amongst which Balkin (2015) highlights four: 1) the more anthropomorphic a robot is, the more people blame the robot, rather than the person who uses it; 2) the presence of robots in a surveillance system increases the subjective feeling that someone is being watched; 3) humans take greater risks to preserve the integrity of anthropomorphic robots than for things designated as tools; and 4) humans may suffer distinct emotional damage from the loss of robotic fellows.

Robotics is no longer restricted to the factory and the laboratory. So-called social robots are designed precisely to interact with humans in uncontrolled environments. To this end, studies and projects have been intensified to develop artefacts capable of interacting with people as naturally as possible. Social robots are characterized by the possibility, albeit apparent, to transmit emotions, encourage and form social relationships, demonstrate personality, use natural clues of communication and interact socially with people. There is already a particular field of study called human-robot interaction (HRI), which seeks, based on social valence, to replicate in robotic artefacts a variety of cues and markers present in human communication, such as facial expressions and even language.

Along with social robots, assistive and rehabilitation Robotics also stand out. Pearl, the Nursebot, is a prototype of a personal mobile robotic assistant that can recognize speech, accompany patients and communicate via touch screen. Designed at Carnegie Mellon University, the nurse robot is being prepared to remind people to take their medicine and help them move around in old people’s homes. Rehabilitation robots were initially designed to assist in the movement of patients in recovery. Assistive Robotics has always had a wide reach, including rehabilitation robots, wheelchair robots, companion robots and manipulative arms. We can also speak of a Socially Assisted Robotics, a term used to describe artefacts whose central focus, instead of physical contact, is some form of social interaction. Robots are already used to help stroke (CVA) patients to do their exercises, to assist the elderly and to care for and educate children and adolescents, especially in cases of specific conditions, as has been advocated in situations of autism.

According to Sharkey and Sharkey (2008), there are several ethical problems related to the use of social robots by people in vulnerable situations. With regard to the elderly, the following are noteworthy: 1) potential reduction in human contact; 2) increased sense of objectification and loss of control; 3) loss of privacy; 4) loss of personal freedom; 5) deceit and infantilisation; 6) uncertainty regarding the circumstances in which the elderly can and should have permission to control robots. For Sparrow and Sparrow (2006), the use of social robots with the elderly reveals a serious ethical problem, as it is based, mainly in the case of anthropomorphic artefacts, on the illusion of genuine social interaction. Even in the case of relatively simple assistive robots, introduced in old people’s homes to monitor their behaviour, one can speak of a technology that decisively affects the choices of these people, which can result in authoritarian Robotics.

When we think of robots as if they were people, we envisage for the artefact a degree of agency and autonomy that is not simply exaggerated, it is actually a transference, in which we lose part of our own autonomy. The proposal of an electronic personhood does nothing to help deal with this problem. It may, in fact, aggravate it, since, even if it is restricted to Law, legal personhood reinforces the concealed equivalence that is symbolically projected towards other fields. But if we move the artefacts away from the idea of natural person, would we not run the risk of abandoning our own ethics in these interactions, as can be seen, for example, with the advance of sexual robots that reproduce misogynistic stereotypes present in society? The social valence of robots shows us exactly the opposite, that is, that ethics can and must precede the definition of the nature of these technologies, by the simple fact that we are human beings, “with autonomy and moral rules, dealing with these ontologically indefinite artefacts” (Cortese, 2018).

A virtue ethics approach can thus offer an interesting way of dealing with the problems generated by the interaction between humans and social robots. To avoid the risk of an individualist solution, Coeckelbergh (2010, 2020) highlights the importance of thinking about a relational and socially oriented ethics of virtue, that is, “virtue in its history and in its concrete bodily performances” (Coeckelbergh, 2020).

The “individualist solutions”, which also mark the philosophical discourse of modernity, have also been transposed to legal discourse. The emphasis placed on the subjective centre of abstract imputation stems from the transposition of an illusion: the individual-subject of law with all his attributes would be capable of shaping the whole juridical system (Alcaro, 1976). While, on the philosophical level, the philosophy of conscience favoured the immediacy of subjective experience over discursive mediation (Habermas, 2007), on the juridical level, processes of social interaction, such as the union of persons around a certain initiative, also came to be portrayed by the interposition of a transcendental subjectivity: the legal person.

## Electronic Persons as Legal Entities

### Legal Entity and Calculation With Concepts

The main argument for the defence of electronic personhood is associated with a pragmatic or functional analysis of legal personhood. In the verbalized legal world, the term “legal person” refers to an autonomous centre of legal relations. If legal personhood is already dissociated from the human substrate, there would be no way to deny personhood to robots due to the non-existence of any human characteristic in these artefacts. In that narrative, the legal person is presented as if there were no problems in the process of attributing legal personhood to companies. The analogy with the legal person requires, however, an understanding of the function of this term in legal grammar.

The philosophical discourse of modernity is not structured only in subjectivity. The rationalization that crystallizes around the organization of the capitalist enterprise and the bureaucratic apparatus of the state also appears as an essential characteristic of those “new times”, with the institutionalization of economic and administrative action with regard to the aims. Law is also going through a process of rationalization, the central idea of which is the differentiation and institutionalisation of autonomous social systems, thought of as machines, since they are founded on themselves and governed by a particular procedural reason. The consolidation of this formal law is not limited to the external foresight of the administration of justice or to the separation of powers, but also requires an internal, predictable control, embodied in the idea that it is “calculated with concepts”, as in mathematics.

The term legal person was perfectly suited to the context of formal Law internally controllable by means of abstract concepts. Even today, when we perceive that this pretension of a legal machine has always been illusory and Law is incalculable, as Irti (2018) pointed out, we can also see that the legal person retains, to a certain extent, its original inspiration: calculation mediated by concepts.

### Functions and Illusions of the Legal Person

According to Solaiman (2017), being a legal person entails the ability to exercise rights and to perform duties. For Bryson et al. (2017), there are three issues related to legal personality that directly interest the debate on electronic personality. First, legal personality is a fiction. Legal personality is not necessarily correlated with an ethical notion of moral personhood. Second, legal personality is divisible. A legal system might treat differently legal entities in respect of some rights and some obligations. Third, the rights and obligations that a legal person may have as a matter of law may not match those it has as a matter of fact (Bryson, 2018). Even agreeing with the points presented, we believe that the heuristic function of the term legal person has a decisive role in the analysis of the proposal to create an electronic personality.

The legal person represents a mental shortcut, a trigger that facilitates access to a set of complex situations. The acts performed by shareholders and directors are unified around abstract subjectivity, and there is no need, in each situation, to refer to the whole set of people who are contemplated by the legal entity’s particular framework. In this sense, it is important to perceive the heuristic function of the term legal person, that is, a mental shortcut that enables, with simplified information, rapid judgements.

As a mental shortcut, legal personhood allows the allocation of the patrimony in autonomous centres, different from the complex of legal relations of each partner. The creation of the new subject (legal person) facilitates the understanding of the separation of assets according to a particular purpose. This, however, creates the illusion that patrimonial segregation is dependent on legal personhood, as if patrimonial autonomy could only be explained with the mediation of the legal person. In addition to the simplification of the complex of relationships and the autonomous allocation of assets, recourse to corporation personhood also allows access to a model of private imputation of acts practiced by shareholders and directors and, at the same time, gives stability to the model of coordination that develops within the legal person.

In the debate on electronic personhood, the process of conferring legal personhood on companies is presented as a model that would justify the recognition of legal personality for robots with artificial intelligence, as argued, for example, by Turner (2018), who even maintains that possible abuses, such as the lack of accountability of programmers and engineers, could be fought by disregarding legal personhood (“piercing the corporate veil”). This type of argument demonstrates how the analogy with corporate law is mobilized without, for this purpose, pointing out the problems present in the model of the corporate personality.

As Galgano (2010) had already reported in Italian law, there are several disadvantages in the process of conferring legal personhood on companies, which are not, to this day, properly measured. Galgano (2010) pointed out that the term legal person was used, both by courts and lawyers, as if there was a single entity to be protected behind the label of the legal person. This form of approach generated a serious problem: unitary treatment. Besides distorting the function of the institute, it masked the diversity of phenomena that articulated around that term. Similarly, Ferro-Luzzi (2001) demonstrated how the idea of activity, fundamental to the understanding of the term enterprise, was mistakenly absorbed by the notion of abstract subjectivity, which, in turn, compromised the very regulation of the business phenomenon by the law. According to the Italian author, the concept of activity depends on a new legal grammar, which reveals itself capable of culturally disassociating the action from the figure of the abstract subject that has rights and duties.

The model of the corporate personality has also contributed to an improper understanding of the limitation of the shareholder’s liability by concealing the unequal transfer of entrepreneurial risk to third parties. If, on the one hand, there are creditors who can protect their own interests by renegotiating the risk with the company, as happens with a financial institution; there are, on the other hand, creditors who are unable to do so, as is sometimes seen with victims of environmental damage, such as those affected by mining. The prevalence of the abstract model of subjectivity has given rise to a unitary reading of patrimonial autonomy itself and, consequently, of the limitation of responsibility, which are indifferent to the different credits.

If the electronic personhood has been conceived according to the problems generated by the need to be accountable for possible damages, it should be remembered that there is a mismatch between the legal format of the isolated corporation and the economic protagonism of the multinational enterprise groups. This is an internal contradiction of Law, materialized in the paradoxical tension between legal diversity and economic unity. To minimize this problem, Law has sought a new grammar, coming closer to the figure of control and direction, breaking with the model of an abstract subject as the central point in the process of accountability.

The creation of an electronic personhood may end up repeating the same problems. Instead of recognizing the peculiarities of the different areas of operation of robots, these different relationships are unified in a single legal model, based exclusively on the figure of an abstract subject. This is a frequent mistake when the law tries to approach new technologies. Instead of their ownership, the artefacts are in fact determined by their specific destinies. Thus, they do not include abstract generalizations and unitary reductions, regardless of their various uses. Is it possible to compare the problems caused by the use of Robotics in medicine with the use of drones for military and security purposes? Similarly, the use of social robots with vulnerable people raises specific ethical problems, which cannot be compared with the use of Robotics for the transport of goods and people.

Accountability focused on the personhood of this new subject, supported by a still debatable concept of autonomy, may conceal those who are truly responsible for the damage and for the development of the artefacts, transferring the risks of the activity carried out by programmers and computer engineers to third parties who share the same spaces with the robots. Contrary to what Turner (2018) states, “piercing the corporate veil doctrine” (disregard of legal entity) does not represent an adequate instrument to remedy these problems, but represents, in fact, a technique that is the main manifestation of the unitarianism that marks the whole discourse of the legal person. There can be seen in the European Parliament’s particular Resolution with recommendations on Civil Law on Robotics, confusion between the attribution of personhood and the separation of patrimony. The creation of a specific fund for any damage caused does not depend on the creation of a new subject, since the legal person, even if associated with patrimonial autonomy, does not have a monopoly on the disposition of property. Nor does criticism of the personification make the disposition of property the main solution to the problem. It is fundamental to come up with differentiated liability mechanisms, sensitive to the different uses of robotic artefacts and the diverse types of damage that may possibly be caused.

On April 21, 2021, the European Commission presented the Proposal for a Regulation on Artificial Intelligence, which seeks to establish a uniform legal framework for the development, commercialization, and use of artificial intelligence within the scope of the European Union. The current proposal moved away from the creation of an electronic legal personality. The text relies on a risk-based approach, which modulates the content of standards according to the intensity of risks created by AI systems.

### Taking Metaphors Seriously: New Subjects and the “Imitation Game”

The proposal to create an electronic personhood is part of a wider debate: the recognition of new subjectivities and, consequently, new legal actors (Gellers, 2020). Teubner (2006) recalls that in 1,522 rats were submitted to a trial in the ecclesiastical court of Autun. The methodological individualism that has informed legal personhood since modern times has prevented the recognition of animal rights. Influenced by the process of rationalization of science and nature, the number of actors in the legal world was, as the German author maintains, drastically reduced by a development of the philosophical discourse of modernity. In dialogue with Luhmann’s Theory of Systems and with Latour’s sociology, Teubner (2006) rejects the anthropocentrism that underlies the psychological and sociological analysis of an intentional action in which the only plausible actor is the human individual.

In 2017, a river in New Zealand was given legal personhood. In the same year, in India, a court recognized the legal personhood of the rivers Ganges and Yamuna. Unlike the Indian case and the New Zealand case, the Constitution of Ecuador made a more daring proposal. The projection of the rights of nature was presented as a way of trying to move from an anthropocentrism to a biocentrism based on the idea of good living. This openness to new forms of subjectivity has the merit of trying to dissociate oneself from the individualistic model that underlies both the natural person and the legal person. But might it be possible to combat anthropocentrism by making use of an instrument such as the legal personhood, the main representative of methodological individualism in legal grammar? Even if these initiatives are of great importance, in a symbolic and cultural dimension, by recognising the wisdom of traditional and indigenous populations with a new cosmovision, the new personalities may end up imprisoned in an old grammar still inspired by an anthropocentric model, such as the ideas of subjective rights and individual ownership. The same can happen with the supposed electronic personhood. Even if the association with the dichotomy natural person and legal person is avoided, the new subjects are articulated by means of old models, which reinforce the already classic subjective modulation of legal discourse.

In the lesson of Rodotà (2015), the problem lies in the perspective of the very idea of an abstract subject that informs any process of attribution of legal personhood. This construction allowed the juridical discourse to formally liberate the person, artificially detaching him or her from his or her economic, social and natural conditions. As a response to the contempt for the concrete, we note the attempt to reconnect the person, in a material sense, to his or her context, with the reinvention of the person, now socio-environmentally situated and embodied.

The pitfall of the metaphor of the abstract subject is precisely that it tends to merge person and juridical subjectivity by not demonstrating the differences and thus hiding them. In Serick’s classic study (1958), there is reference to the teratological case People’s Pleasure Park Co. v. Rohleder, in which a Virginia court in 1908 asked itself what the colour of the legal person would be when faced with the following question: whether a society, as an autonomous centre of juridical relations, could be constrained by the racist laws of the state which prohibited blacks from acquiring land. In Germany, with the rise of Nazism, the courts also had to examine whether the anti-Semitic laws could be applied to companies controlled by Jews (Serick, 1958).

In the case Santa Clara County v. Southern Pacific Railroad, the term “person”, provided for in the 14th amendment of the US Constitution, was also associated with a corporation, which could be seen as an example of a subject for Law (Hall, 2005). In 2014, in a controversial decision, the US Supreme Court resorted to the argument that an entrepreneurial society, Hobby Lobby, could invoke religious freedom in order not to collaborate with the payment of a health plan that would allow employees access to emergency contraceptive drugs, with high doses of oestrogen, popularly known as morning-after pills.

The accommodation of the religious freedom of a for-profit business society comes up against an important point, however: thousands of women employed by Hobby Lobby may not share the same belief as the main shareholders in the company. In view of this situation, did the court decide to protect the legal position of the company’s controlling shareholders to the detriment of the private autonomy of the female employees? For Judge Ginsburg, the casting vote at the time of the trial, there was no doubt: the choice to extend religious freedom to a profit-making organisation generated a serious imbalance within the company by favouring the belief of the controllers over the protection of the rights of women working in the company in question.

In the debate on the rights of the personhood of legal persons and on the moral damage to legal persons in Brazil, there is a sometimes problematic approach between natural persons and legal persons. This equalisation may, as already highlighted, ignore the diversity of interests that justified the personification of the human being in relation to the embodiment of companies, foundations and associations. Just as it is important to criticize the disguised fusion between person and legal person, we should also separate person and legal personhood and recognize that the expansion of new subjects refers only to the latter, to juridical subjectivity.

In this context of new subjectivities, what should be done? Albeit controversial, the very origin of the term legal personhood, derived from the term *persona*, is associated with a metaphor, the mask used in theatre, allowing the actor to impose his voice. Despite this remote use, people still believe today in the illusory possibility that metaphors, even those already incorporated within legal grammar, can be prohibited. Italian nominalism, recognising that the legal person would represent a linguistic instrument, almost suggested its end, thus underestimating the power and function of metaphors. Even if there is no way to eliminate them, it will always be possible to monitor their normative use, reporting, in specific situations, the abuses related to the use of metaphors and analogies in a literal sense.

As Turner (2018), one of the enthusiasts of the attribution of legal personhood to robots, points out, the accreditation of electronic personhood to robots in the United States or the European Union is likely to influence other legal decisions. The electronic personhood may thus be adopted by countries that traditionally import legal models, as is the case of Brazil, whose model of legal personhood for natural persons has never been fully achieved. Political, economic and social challenges have prevented, and still prevent, the construction of a complete citizenship in several peripheral countries. Although influenced by the philosophical discourse of modernity, the adoption of legal models in Brazil has occurred, in various situations, in a particular and partial way, as in a real game of imitation, an incomplete and untimely simulacrum of never-realised expectations. We cannot move on to new subjectivities without confronting old promises, such as the problems of subjects whose human rights have not yet been achieved, at the risk of confusing people and legal entities. Perhaps robots with artificial intelligence can wait for their controversial rights. Perhaps the only task, no less important, left for us to carry out is that of adjusting subjects, putting back on the masks and taking the metaphors seriously, that is, continuing to report the non-problematised convergence between the contemplated metaphor and the disguised comparison.

## Conclusion

The sentence “the robots are coming”, which has already become a cliché, does not accurately portray the evolution of this technology. If robots, in fact, have already arrived, what is this so loudly proclaimed Robotics revolution? Robotic artefacts, in contrast to what used to happen, are increasingly integrated into the same environments as human beings, which, in turn, can have great impacts, not yet fully measured, as can be seen in the use of these technologies in medical care and care for the elderly and children. The imaginary about robots is intensely marked by the association with anthropomorphic artefacts, such as androids, which appear in films and literature. A particularly dangerous metaphor for the law is to yield to this symbolism, projecting autonomy, consciousness and other human attributes into robotic artefacts. Often the different concepts, originally fused around the metaphor, disappear, so that differences are erased and metaphors and analogies come to life, coming to be thought of in their literal sense.

The discussion about the ontological foundations that separate people and robots has been seen to be insufficient to remove the defence of legal personhood from robotic artefacts with artificial intelligence. If the law confers legal personhood on assets intended for certain purposes, such as foundations, there can be no doubt that the aptitude to acquire rights and duties is not exclusively one of human beings. In fact, we note the prevalence of a pragmatic or functional line of the electronic personhood, which, by distancing itself from the philosophical debate centred on ontological analyses, seeks to base itself mainly on the model of the corporate legal personality. This change of focus, with robots as legal persons, also involves problems, which in most cases are neglected even by critics of the electronic personhood. This is mainly on account of the incorrect understanding of the reasons present in the process of embodiment of companies and the particular role of the term “legal person” in the grammar of Law.

In Jorge Luis Borges’ fictional essay, the substitution of the anthropomorphic metaphor by a sphere inspired several thinkers, until it became a labyrinth and an abyss for Pascal, who, feeling the incessant weight of the physical world, adjusted his metaphor, going on to claim that “nature is an infinite sphere, whose centre is everywhere and its circumference nowhere”. Blaise Pascal, whose studies were fundamental for computing, was also known for his wager as to the infinite. In this single player game, we can reflect ethically on the existence of the indefinite, even if it is rationally inaccessible. In the same way, we do not need to wait for ontological definitions or these robotic artefacts to definitively become part of people’s everyday lives to question ethical problems related to this process. Should we be concerned about social robots? What are the main risks associated with the so-called Socially Assistive Robotics? If, on the one hand, the electronic personhood contributes very little to the problems generated by the not at all metaphorical approximation between robots and humans; on the other hand it reinforces dangerously the connection, not always questioned, between anthropomorphic rhetoric and concealed imitation.

## Data Availability

The original contributions presented in the study are included in the article/Supplementary Material, further inquiries can be directed to the corresponding author.
